# Electron Imaging of Nanoscale Charge Distributions Induced by Femtosecond Light Pulses

**DOI:** 10.1021/acs.nanolett.4c00773

**Published:** 2024-05-03

**Authors:** Jonathan T. Weber, Sascha Schäfer

**Affiliations:** † Institute of Physics, Carl-von-Ossietzky University of Oldenburg, 26129 Oldenburg, Germany; ‡ Department of Physics, University of Regensburg, 93053 Regensburg, Germany; ¶ Regensburg Center for Ultrafast Nanoscopy (RUN), University of Regensburg, 93053 Regensburg, Germany

**Keywords:** ultrafast transmission electron microscopy, nonlinear photoemission, gold nanostructures, Lorentz microscopy, photovoltage

## Abstract

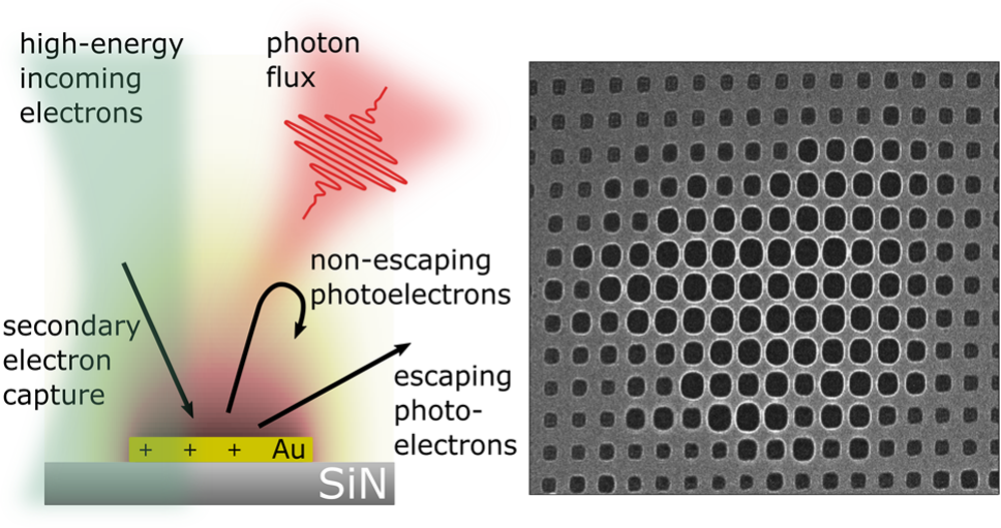

Surface charging is ubiquitously observable during in situ transmission electron microscopy of nonconducting specimens as a result of electron beam/sample interactions or optical stimuli and often limits the achievable image stability and spatial or spectral resolution. Here, we report on the electron-optical imaging of surface charging on a nanostructured surface following femtosecond multiphoton photoemission. By quantitatively extracting the light-induced electrostatic potential and studying the charging dynamics on relevant time scales, we gain insights into the details of the multiphoton photoemission process in the presence of an electrostatic background field. We study the interaction of the charge distribution with the high-energy electron beam and secondary electrons and propose a simple model to describe the interplay of electron- and light-induced processes. In addition, we demonstrate how to mitigate sample charging by simultaneously optically illuminating the sample.

Sample charging in electron microscopy results from a number of interlinked interactions between high-energy electrons and nanoscale specimens, such as electronic excitation, defect generation, and the emission of secondary electrons.^1^

For example, in cryo-electron microscopy, the interaction of imaging electrons with accumulated charges in amorphous ice films is often detrimental and poses a limit to the achievable spatial resolution, image stability, and image contrast.^2−5^ An ongoing effort is made to quantify and understand the contributing processes in detail^6−8^ as well as to mitigate the adverse effects of sample charging.^9,10^

While in many cases sample charging needs to be minimized, other fields like liquid phase electron microscopy^11^ often rely heavily on the interaction of the sample with the electron beam, utilizing the charge accumulation for the electron-beam-induced fragmentation of precursors^12,13^ or charging-induced ion transport.^14−16^

Despite the distinct underlying mechanisms, sample charging in weakly conducting specimens is also commonly encountered in photoemission spectroscopy and microscopy approaches.^17−19^ Here, light-induced surface charging manifests as a shift and, for inhomogeneous charging, as a broadening in the measured photoelectron spectra as well as a suppression of the total photoelectron yield. In some cases, sample charging can be counteracted by the use of an additional low-energy electron beam neutralizing the charge distribution.^20,21^

Similarly, in the emerging field of electron microscopy with in situ optical excitation^22−25^ and ultrafast transmission electron microscopy,^26−36^ sample charging is expected to simultaneously occur due to light- and electron-beam-driven processes. Fully understanding the effects contributing to charge accumulation in these systems necessitates experiments that address the sample response to optical and high-energy electron stimuli as well as the interplay of these effects.

Here, we report on the light-induced charging of individual gold nanostructures, imaged via transmission electron microscopy (TEM). The induced photovoltages are quantitatively extracted by comparing the defocused experimental electron micrographs to electron-optical image simulations, using a numerically calculated electric potential distribution. The effective nonlinearity of the underlying photoemission process is precisely measured using interferometrically stable two-pulse excitation and event-based electron detection, gaining insight into the interplay of light- and electron-beam-induced charging phenomena and their significance for photoemission processes in electron microscopy with in situ optical excitation.

To investigate light-induced charging in electron microscopy, we consider arrays of gold nanoislands on an insulating silicon nitride membrane as a model system (see Methods for details). Using the Oldenburg ultrafast transmisison electron microscope (UTEM), we illuminate the sample in situ with femtosecond optical pulses (800 nm central wavelength, 169 fs pulse duration, illumination area widened to a diameter of 30 μm, 400 kHz repetition rate, p-polarized). The photon energy is deliberately chosen to be below the workfunction so that linear photoemission processes are excluded. For mapping the temporally averaged charge state of individual islands, we employ a continuous electron beam and large electron imaging defoci of −10.5 mm. A typical defocused micrograph of a gold island without optical illumination is shown in Figure 1b. Upon illumination (1.2 mW average optical power), a drastic change in image contrast occurs (see Figure 1c), which results in an increase in the apparent nanostructure size by a factor of 1.4 and a change in the electron interference pattern around the nanodisc. Different islands within the illuminated part of the array show comparable light-induced contrast changes with only minor variations, as shown in Figure 1a (bottom micrograph).

**Figure 1:**
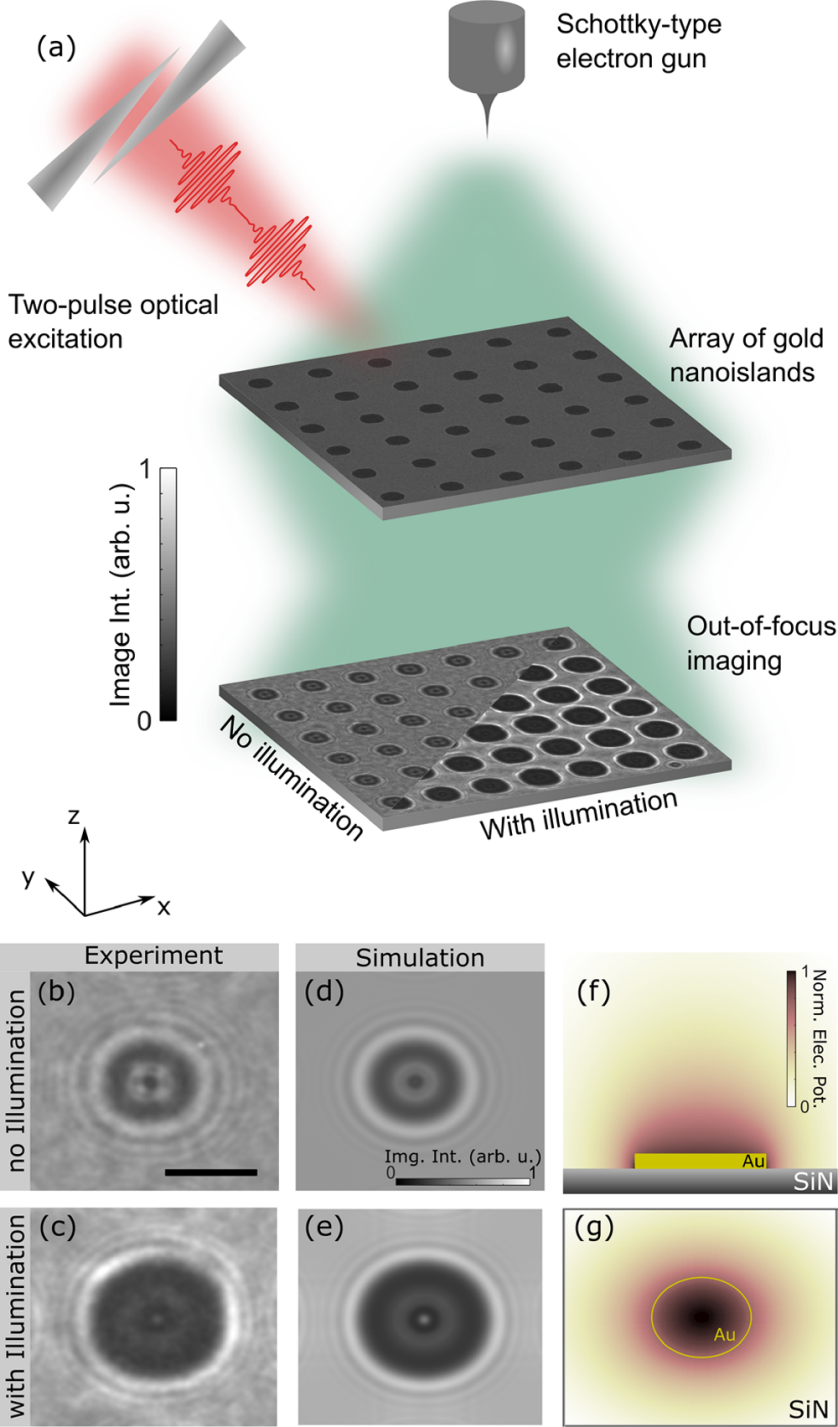
Electron imaging of light-induced charging in a transmission electron microscope. (a) Scheme of the experimental setup. Optically induced charging of isolated metallic islands in the sample plane results in strong contrast modulations in the image plane under out-of-focus imaging conditions. (b and c) Experimental Lorentz micrographs without and with illumination, respectively, are compared to image simulations (d and e). The scale bar is 500 nm. The spatial electric potential distribution used in the image simulations is numerically calculated by employing a successive over-relaxation approach. Summed distribution shown in (f) side view and (g) top view, with the sample structure indicated by the sketch (not to scale).

Using an electron-optical simulation,^37^ the image contrast can be quantitatively reproduced considering an optically induced positive charging of the gold island to a photopotential of 3.9 V (Figure 1d,e), corresponding to a charge depletion by ∼500 electrons per island. The electrostatic potential distribution surrounding the metallic island was determined by numerically solving the electrostatic Laplace equation, using a three-dimensional successive over-relaxation method^38^ and cross-checked with a commercial finite-element simulation software (Figure 1f,g). The electric potential imprints a phase shift onto the imaging electron beam,^39^ which translates into an image contrast under out-of-focus conditions.^37^ For a quantitative determination of the induced photovoltage with varying optical excitation, the image intensity is fitted by minimizing the squared differences between experimental and simulated image intensities, with the light-induced voltage *U*_PV_ on the metallic islands as the only free fitting parameter. We attribute the observed charging to a multiphoton photoemission process facilitated by the high intensities of the femtosecond light pulses (estimated peak intensity of 3.1 GW/cm^2^). We note that our experimental conditions, with a substrate with low electric conductivity, are tuned to achieve a long lifetime of the charge-depleted state, so that the final state after femtosecond charging can be studied with a continuous electron beam.

To further characterize the multiphoton photoemission process, we conducted experiments with phase-stable pairs of collinear optical pulses with an adjustable delay. For this purpose, a birefringent common-path interferometer was introduced into the optical-beam path, similar to a translating-wedge-based identical pulses encoding system (TWINS).^40^ For pulse delays smaller than the temporal pulse widths, the interferometer modulates the overall optical power due to the interference of both pulses, with the modulation period given by the optical period of the light pulses.

At larger pulse-to-pulse delays, the interferometer can be utilized to investigate potential non-instantaneous light-induced dynamics. Thus, the control of the optical fluence and the study of transient effects are conducted with the same setup, ensuring high comparability throughout the different experiments. Experimentally, we observe that the defocused micrographs strongly depend on the pulse delay. In Figure 2a, image intensity profiles across a single disc are shown for varying pulse delays close to zero. The corresponding recorded optical power (blue line) and extracted photovoltage (red circles) are displayed in Figure 2b (see also Movie 1). Whereas the optical power exhibits a simple harmonic dependency on pulse delay as expected, both the experimental profile widths and the extracted photovoltage show a more complex behavior. We accumulate the data from the four optical cycles shown in Figure 2b and plot the photovoltage depending on the light intensity (Figure 2c), confirming that for these delays the photovoltage is given as a function of the optical power. In a logarithmic plot, the power scaling of the photovoltage at a low fluence shows a slope of 6.5, which is higher than the expected value of 4, given the photon energy of 1.55 eV and gold’s workfunction of ∼5.3 eV.^41^ At higher light intensities, the photovoltage saturates. A potential explanation could be space charge effects within a photoemitted electron cloud.^42−44^ However, as detailed below, for our system this behavior is linked to the cumulative charging of the sample over successive light pulses. We note that beyond driving the nonlinear photoemission, the optical excitation also results in an increased base temperature of the sample, influencing to some extent the photoelectron yield and substrate conductivity. Comparing the employed optical pulse fluences of ≤0.5 mJ/cm^2^ to those of previous experiments on a similar sample system,^45^ we estimate the average temperature increase in our case to be <50 K.

**Figure 2:**
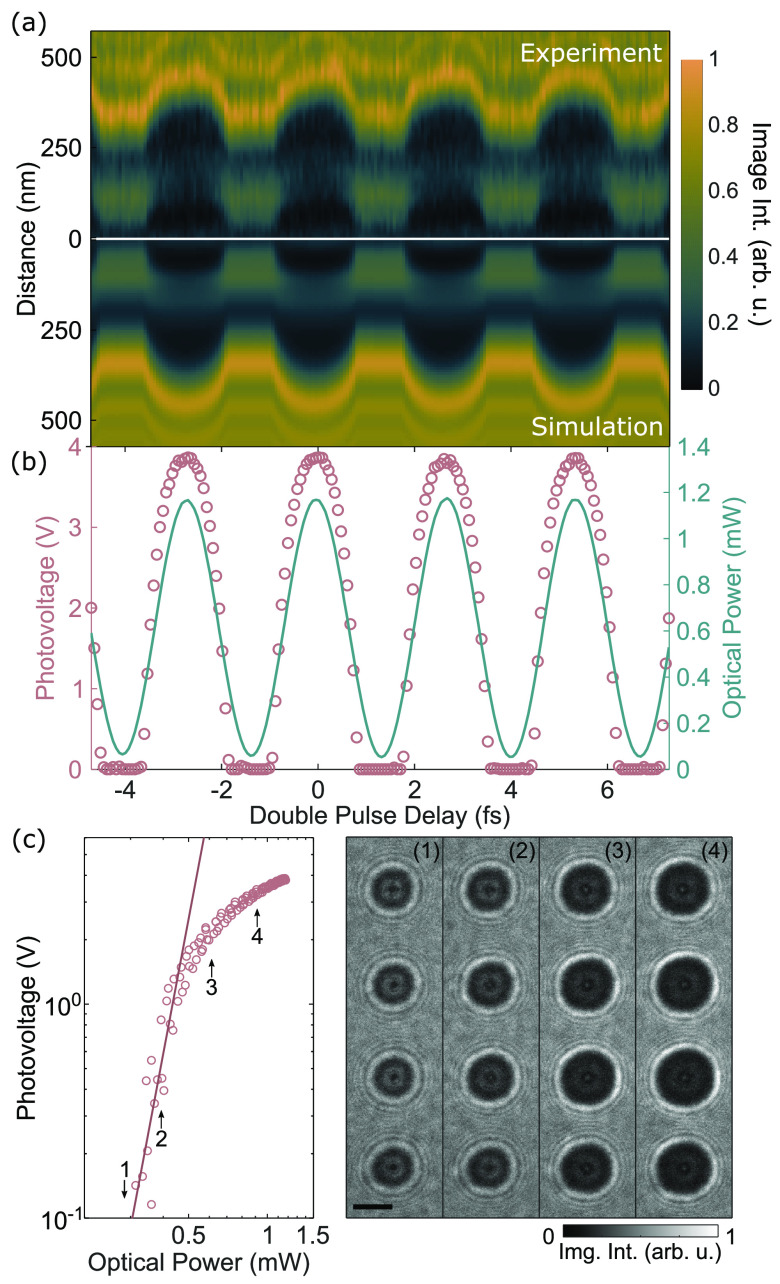
Interferometric two-pulse optical excitation and quantitative determination of the photovoltage. (a) Image intensity profiles through the center of a metallic island, averaged over three pixels, for a respective optical power from experimental micrographs (top) and simulated image intensities (bottom). (b) Incident optical power (blue line) during an interferometric two-pulse excitation measurement in the vicinity of zero pulse-to-pulse delay. The optically induced voltage on a metallic island (pink circles) is determined by nonlinear least-squares fitting of the image simulation to experimental micrographs with the photovoltage as the only free parameter. (c) Light-induced voltage on a metallic island as a function of optical power. The straight line represents a slope of 6.5 in the logarithmic plot. The right panel shows experimental micrographs of a column of gold islands at the indicated optical power. The scale bar is 500 nm.

We further investigated the induced photovoltage for larger pulse delays (see Figure 3a). The optical power variation traces the field autocorrelation of the optical excitation pulse (spectral width of ∼6.8 nm). Due to the nonlinear intensity dependence mentioned above, the photovoltage shows a distinctly different behavior compared to the autocorrelation but without a clear signature of a delayed sample response, for example, due to a hot electron gas.

**Figure 3:**
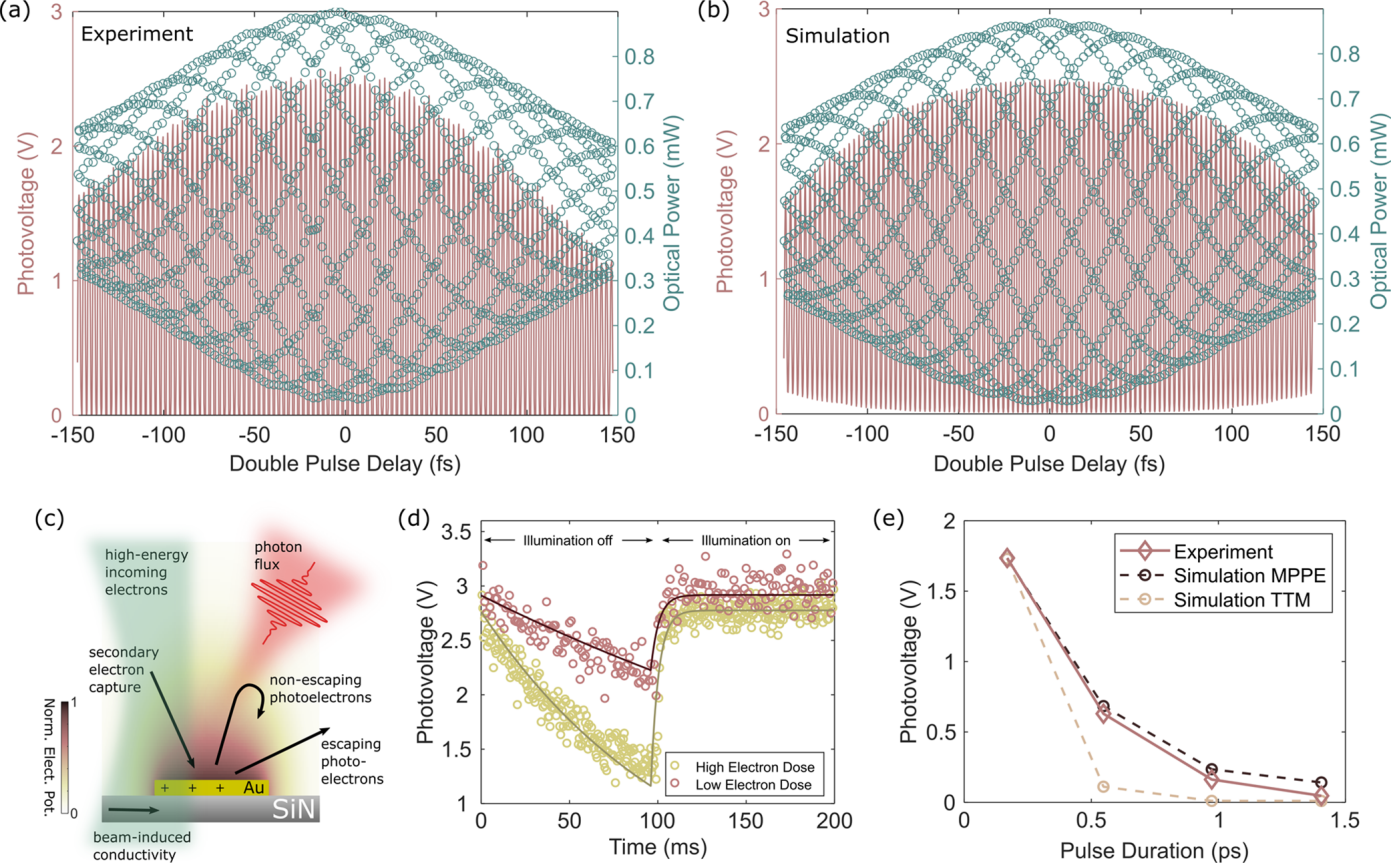
Dynamics of the charging/decharging process and interplay of light- and electron-beam-induced phenomena. (a) Photovoltage (pink line) during interferometric double-pulse optical excitation for pulse delays of up to ±150 fs. The measurement of the optical power (blue circles) corresponds to the field autocorrelation function. (b) Simulation of the photovoltage for varying pulse-to-pulse delays of the optical excitation in an interferometric measurement scheme. (c) Overview of the electron-beam- and light-induced processes contributing to the charging/decharging of individual metallic islands. (d) Charging/decharging cycle measured with an event-based electron detector for an electron dose rate of 0.012 electron nm^–2^ s^–1^ (pink circles) and 0.043 electron nm^–2^ s^–1^ (yellow circles). The dynamics are modeled with a rate equation (dark red and yellow lines) including optical- and electron-beam-induced contributions to the charge state of the islands (see the text for details). (e) Experimentally measured photovoltages for optical excitation with varying pulse lengths and comparison to the simulated results considering a multiphoton photoemission process and a thermionic emission process. Simulations are adjusted to replicate the experimental photovoltage for unstretched light pulses.

To understand the apparent saturation of the photoemission efficiency at higher fluences, we apply an electron microscopy approach with high temporal resolution and investigate the cumulative charging over successive optical pulses by using an event-based electron detector based on a TimePix3 chip architecture.^46−48^ Using a Pockels cell, we precisely chop the optical excitation at a frequency of 5 Hz (50% duty cycle) and collect an 8 s electron event stream on the TimePix detector. The events are sorted into 500 μs wide bins according to their relative delay to the 5 Hz control signal. The photovoltages extracted from these reconstructed micrographs (see also Movie 2 and Movie 3) are shown in Figure 3d for electron-beam dose rates of 0.012 electron nm^–2^ s^–1^ (pink circles) and 0.043 electron nm^–2^ s^–1^ (yellow circles).

For delays from 0 to 100 ms, no illumination of the sample occurs and a decharging of the metallic islands can be observed. This process is governed by the emission of secondary electrons in the vicinity of the charged islands by the incident high-energy electron beam, which neutralize the positively charged metallic nanostructures.^2^ Generally, the number of emitted secondary electrons depends on the electron dose and the substrate material. In our case, part of the silicon frame that holds the silicon nitride membrane is iluminated by the electron beam, resulting in a higher secondary electron yield. Consequently, different decharging rates of 8 and 21 V/s are observed for the different electron-beam dose rates. A further contributing charging mechanism might be an electron-beam-induced increase in the electrical conductivity of the silicon nitride substrate. We note that the gold islands themselves can also be considered as a source of secondary electrons, albeit with a much weaker influence in comparison to the dominant contribution of the 200 μm thick silicon frame.

Upon reillumination of the sample at a delay of 100 ms, a fast increase in the photovoltage can be observed, with a time constant of ∼2 ms. Subsequently, after the cumulative effect of ∼10^3^ optical pulses, the measured photovoltage remains at a constant *U*_PV_ for the duration of the optical excitation.

The observed behavior can be explained within the framework of photoemission in a background electric field. Photoemitted electrons have to overcome the electrostatic potential that surrounds the charged islands. As a consequence, electrons with insufficient initial energy from the multiphoton absorption are unable to escape the potential well and instead fall back to the surface of the metallic island, not contributing to a further charging of the island. At a saturated photovoltage (at delays >100 ms), only a few photoelectrons escape the Coulomb potential around the islands, that balance the electron-beam-induced decharging, thereby maintaining an equilibrium potential state of the island. Supporting this picture, TimePix-based recordings with nanosecond time bins (see Movie 4) did not show any apparent contrast changes between optical pulses, highlighting that in the saturated state, only minimal charging occurs. The contributing processes are sketched in Figure 3c.

Along these lines, also the observed distinctive intensity dependence, characterized by a consistent decrease in slope as the optical power increases, can be explained. We describe the dynamic charging process with a rate equation model, in which the change in photovoltage *U*_PV_ of an island is given by
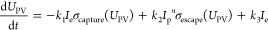
1The first part of the expression corresponds to the decharging of the metallic nanoisland due to the capture of secondary electrons induced by the beam current *I*_e_. The capture probability of secondary electrons, σ_capture_(*U*_PV_), depends on the charge state of the islands. For the sake of simplicity, we consider a simple relation σ_capture_(*U*_PV_) = *U*_PV_ that results in an exponential decay of the island’s charge state. The second term describes the charging of the islands due to photoemission and depends on the optical intensity *I*_p_, the effective nonlinearity of the photoemission process *n* and the probability σ_escape_(*U*_PV_) that a photoemitted electron escapes the electrostatic potential of the charged island. The third term represents the contribution of the electron-beam-induced positive charging to the overall dynamics due to the emission of secondary electrons from the nano disc. *k*_1_, *k*_2_ and *k*_3_ are rate constants of the involved processes. The escape probability can be approximately connected to the photoelectron energy distribution *g*(*E*), yielding
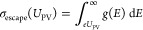
2In general, *g*(*E*) will change with the optical intensity due to the opening of multiphoton emission channels with higher nonlinearities at increased intensities. By using a constant photoelectron energy distribution with an upper limit of 2.99 eV, we already obtain a good fit to the experimental time-resolved photovoltage traces, as shown in Figure 3d. With the same set of fit parameters, also the absolute value of the photovoltage induced by double-pulse excitation can be well reproduced (Figure 3a,b), despite the different electron-beam dose rates and temporal optical pulse shapes employed for these experiments. Minor misalignments in the common-path interferometer lead to a nonvanishing fluence for destructive interference around zero delay. This is taken into account in the simulations by assigning slightly different amplitudes to the electric fields of the two pulse copies.

To clarify the characteristics of the photoemission process, we further conducted measurements with varying optical pulse lengths (see Figure 3e). We incorporated dense-flint glass cylinders with an effective length of 10 cm (20 cm, 30 cm) into the optical-beam path, thus introducing a strong chirp to the pulses, effectively stretching the optical excitation to a duration of 0.55 ps (0.97 ps, 1.4 ps). A comparison of the experimental data (pink circles) to the photovoltages expected from a pure multiphoton photoemission process in a background field (black cirlces) and a thermionic emission process in a background field (yellow circles), calculated by utilizing a simple two-temperature model, suggests that the multiphoton pathway is dominating at the employed excitation parameters.

Whereas, so far, we have focused on the quantitative description of the light-induced charging and photoemission characteristics, we note that light-driven processes can also be utilized to compensate electron-beam-induced charge accumulation. Under illumination conditions for which the primary beam is not impinging on the silicon support frame of the membrane, secondary electron emission is minimized, and other decharging mechanisms may become relevant. In Figure 4a–d, gold islands are imaged under such conditions and at different light intensities. Without optical illumination (Figure 4a), the image of the island array is strongly distorted and changed in its effective magnification by a factor of 3.5 due to an electron-beam-induced local charge accumulation and thereby the formation of an electrostatic lens. As compared to the laser-induced charging experiments, less contrast is seen around each nanoisland, putatively due to a more homogeneous charge distribution in the electron-beam-induced case. We observe a reduction in the apparent magnification upon illuminating the charged area with femtosecond light pulses, indicating a neutralization of the accumulated electron-beam-induced charge (see Figure 4a–d). The decharging effect becomes more pronounced for higher optical powers (see Figure 4e; optical spot diameter of 10–15 μm) and occurs only when the spatial overlap between the electron beam and the optical focus is maintained.

**Figure 4:**
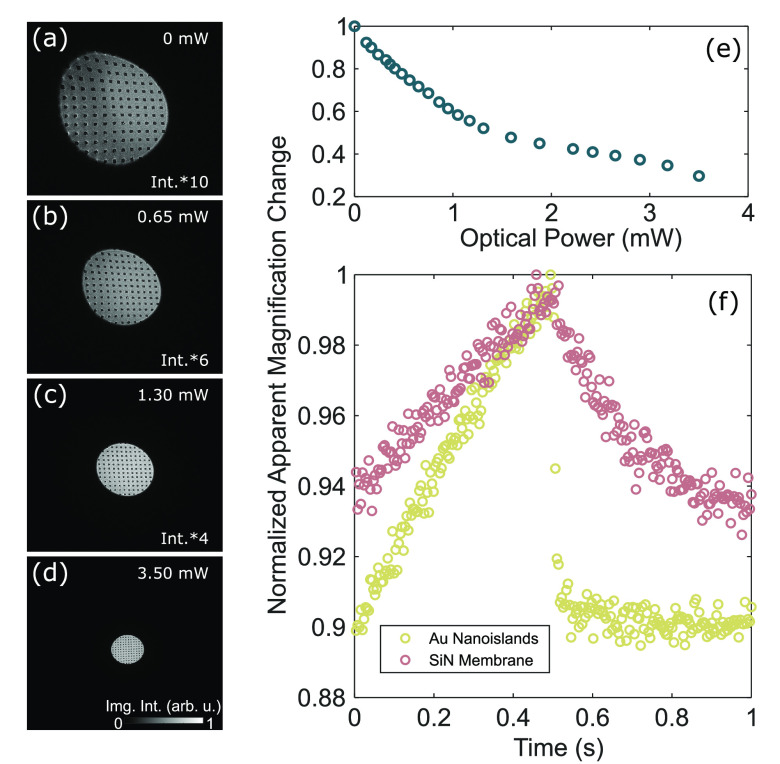
Mitigating electron-beam-induced charging by optical illumination. (a–d) Experimental Lorentz micrographs showing a strong electron-beam-induced change in the apparent magnification and image distortions (electron dose rate of 0.02 electron nm^–2^ s^–1^, defocus value of −10 mm). The sample is illuminated with femtosecond light pulses with the indicated average optical power. The image intensity is multiplied by the indicated factor to increase the contrast. The nanoisland periodicity is 1.5 μm. (e) Change in the apparent magnification for an array of illuminated gold nanoislands as a function of average optical power. The apparent magnification is measured as the Gaussian width of the electron spot along one dimension. (f) Change in the apparent magnification measured with an event-based electron detector while chopping the optical excitation (50% duty cycle, 1 Hz). The decharging dynamics are investigated on an array of gold nanoislands (optical power of 0.8 mW, yellow circles) and an empty silicon nitride membrane (optical power of 2.3 mW, pink circles). Both measurements were conducted with an electron dose rate of 0.012 electron nm^–2^ s^–1^ and a defocus value of −20 mm. We note that the photoinduced magnification changes sensitively depend on the illumination conditions.

We investigate the dynamics of charge buildup and light-induced neutralization using the event-based electron detection scheme, while chopping the optical excitation at a frequency of 1 Hz (50% duty cycle), on an array of gold nanoislands as described above and an empty silicon nitride membrane. For delays between 0 and 500 ms, no optical illumination occurs, and in both samples, an increase in the apparent magnification due to electron-beam-induced charge accumulation can be observed. Reillumination at a delay of 500 ms results in a decrease in the apparent magnification. For the silicon nitride membrane (without gold islands), we observe a progressive decharging for the remainder of the illumination period, just starting to saturate at a delay of 1 s. If additionally gold nanoislands are present in the area under investigation, we observe a step-like compensation of the accumulated charge, reaching an equilibrium state after a few milliseconds.

Optical illumination has been reported to reduce surface charging effects in electron microscopy,^49^ using photon energies close to the workfunction of the materials by detrapping accumulated charges. As a direct excitation of surface defects is expected to be largely suppressed at the wavelength employed in our experiments, we hypothesize that instead the observed behavior could be attributed to an optical-pumping-induced, locally increased sample temperature. The enhanced electrical conductivity would shift the equilibrium point of electron-beam-induced charge accumulation to smaller values. Additionally, internal photoemission processes might contribute to the observed behavior.

We note that optical illumination serves as an effective method for mitigating the detrimental impacts of sample charging in electron microscopy, reducing electron-beam-induced lensing effects by a factor of ≤3 in our experiments. Beyond this application, the strong image contrast modulations upon optical illumination through either charging of individual gold nanoislands or decharging of the sample provide a viable tool for finding the spatial overlap between an electron beam and optical foci on the sample, necessary in optical in situ TEM and in UTEM approaches, which is usually a very time-consuming task requiring meticulous alignment. Furthermore, this technique offers an easy approach for estimating the size of the illuminated area on the sample (see Figure S1 for an electron micrograph of an inhomogeneously illuminated array of square-shaped nanoislands).

In conclusion, we present the electron-optical imaging of light-induced charge distributions on a nanostructured surface. We quantitatively determined the photovoltage by reproducing the experimental micrographs with electron-optical image simulations using a numerically calculated electrostatic potential distribution. By utilizing interferometric two-pulse excitation measurements and event-based electron recording, we could identify the underlying process as a multiphoton photoemission process in a background electric field in the presence of low-energy secondary electrons. We modeled the charging dynamics with a rate equation and quantified the contributions of light- and electron-beam-induced effects. With the same set of parameters, we were able to quantitatively reproduce the observed photovoltages for different electron-beam doses, optical powers, and effective optical pulse lengths, highlighting the quality of our model. In the future, our findings may help to disentangle the various charging-related phenomena and enable a more precise and controlled characterization of nanoscale materials and devices. First results on light-induced decharging processes show potential to mitigate adverse effects of charging dynamics in high-resolution electron microscopy.

## Methods

### Specimen Preparation

The investigated samples consist of an array of disc-shaped Au islands with a diameter of 500 nm and an interisland spacing of 1 μm. Using a lift-off process, the specimens were prepared on 50 nm thick silicon nitride membranes (PELCO) as a substrate. A mask was patterned by electron-beam lithography into a poly(methyl methacrylate) (PMMA) resist with subsequent development. Using electron-beam vapor deposition (base pressure of 10^–7^ mbar), the sample was coated with a 3 nm chromium layer, followed by a 17 nm gold layer. The chromium film acts as a wetting layer for the subsequently evaporated gold, promoting adhesion to the substrate and enabling the lift-off procedure. The interdiffusion and alloy formation of the two metallic layers are limited to a range of 2–3 nm,^50^ so that the influence of the chromium layer is neglected in the interpretation of the photoemission data. The final structure thickness was confirmed by atomic force microscopy measurements.

### Electron Microscopy

Electron micrographs were recorded with the Oldenburg ultrafast transmission electron microscope, which is based on a JEOL JEM-F200 instrument (200 keV electron energy, Schottky-type electron gun). The microscope was operated in the low-magnification mode with the objective lens turned off, and a defocus of −10.5 mm was chosen, unless stated otherwise. For electron illumination, a 100 μm diameter condenser aperture and a spot size of 5 were used. Micrographs were acquired with a complementary metal oxide semiconductor (CMOS) detector (TVIPS TemCam-XF416R, 4096 pixels × 4096 pixels, 15.5 μm pixel size). Matlab was used for all further evaluation steps, which included binning (4 pixels × 4 pixels) and Gauss filtering (standard deviation of the two-dimensional Gaussian smoothing kernel of 2) and analysis of the photovoltage. For time-resolved measurements, we utilized a TimePix3 detector (Cheetah T3, Amsterdam Scientific Instruments), which is an event-based electron detector with a nominal time bin width of 1.6 ns.

### Optical Setup

For triggering multiphoton photoemission from the gold nanoislands, we used optical pulses from a collinear optical parametric amplifier (OPA, Orpheus HP, Light Conversion) seeded and pumped by an amplified Yb-doped potassium gadolinium tungstate (KGW) femtosecond laser system (Carbide, Light Conversion). Optical pulses were characterized by a self-built frequency-resolved optical gating setup (FROG). Femtosecond light pulses are focused on the sample using an incoupling unit installed on a flange located at the height of the TEM pole piece. The incoupling unit consists of a vacuum viewport and a focusing lens (focal length of 50 mm, diameter of 0.5 in.) that is mounted on three piezo stages, enabling high-precision scanning of the optical focus over the sample, with an incident angle of ∼57° relative to the electron beam. An active beam stabilization system (Aligna, TEM Messtechnik) is utilized to accommodate the relative movements of the optical laser system and the TEM column, each supported on individual vibration damping systems.

For the generation of interferometrically stable optical pulse pairs with an adjustable delay, we used a birefringent common-path interferometer.^40^ Specifically, a half-wave plate is used to polarize the light at 45° relative to the fast axis of a planar α-BBO crystal (thickness of 4 mm). The fast axis of the following pair of α-BBO wedges (length of 50 mm, opening angle of 7°) is rotated by 90° with respect to that of the planar α-BBO crystal. The last element of the interferometer is a polarization filter, with the transmission axis at a 45° angle with respect to the fast axes of the planar and the wedged α-BBO crystals.

## Supporting Information Available

Assembled video of the data presented in Figure 2 (Movie 1) (AVI)
Assembled video of the data presented in Figure 3, with a high-dose electron beam (Movie 2) (AVI)
Assembled video of the data presented in Figure 3, with a low-dose electron beam (Movie 3) (AVI)
In situ observation of nanoislands, optically excited with a repetition rate of 400 kHz (Movie 4) (AVI)
Additional details of the data analysis and computational methods, including a micrograph showing an inhomogeneously excited array of nanoislands (PDF)

## Supplementary Material

nl4c00773_si_001.avi

nl4c00773_si_002.avi

nl4c00773_si_003.avi

nl4c00773_si_004.avi
