# Supplementary material for: Electron Imaging of Nanoscale Charge Distributions Induced by Femtosecond Light Pulses
Source: Nano Lett. 2024 May 3;24(19):5746–53. doi: 10.1021/acs.nanolett.4c00773 (PMC11100287; doi:10.1021/acs.nanolett.4c00773)
Supplement: Supplementary file 5 — nl4c00773_si_005.pdf [file nl4c00773_si_005.pdf]

Supporting Information for: Electron Imaging of Nanoscale Charge Distributions Induced by Femtosecond Light Pulses

Jonathan T. Weber[†,‡] and Sascha Schäfer[*,†,‡,¶]

†*Institute of Physics, Carl-von-Ossietzky University of Oldenburg, 26129 Oldenburg, Germany*

‡*Department of Physics, University of Regensburg, 93053 Regensburg, Germany*

¶*Regensburg Center for Ultrafast Nanoscopy (RUN) University of Regensburg, 93053 Regensburg, Germany*

E-mail: sascha.schaefer@ur.de

This PDF file includes:

Additional Information on the image contrast simulation, the electrostatic potential model and the image fit procedure.

Figure S1

Captions for Movies 1-4

Other supporting materials for this manuscript:

Movies 1-4

Image Intensity Calculations

The wavefunction Ψ of the electron wave after passing the sample is spatially phase-modulated by the charged nanoislands and expressed as $\Psi(\vec{r}) = A(\vec{r})e^{i\Phi(\vec{r})}$, with $A(\vec{r})$ and $\Phi(\vec{r})$ being the amplitude and phase imparted on the incident electron plane wave Ψ_0. The vector $\vec{r}$ denotes the position in planes perpendicular to the electron trajectory which is chosen to be the z-direction. For numerical simulations, we discretize $\Psi(\vec{r})$ on a 1024x1024 grid, with an effective pixel spacing of 6.6 nm. The phase shift imprinted on the incident electron wavefront is given by the Aharonov-Bohm phase[1] in the weak-deflection limit

$$\Phi(\vec{r}) = \frac{e}{\hbar v} \int V\left(\vec{r}, z\right) \mathrm{d}z. \tag{1}$$

Here, $V\left(\vec{r}, z\right)$ is the electrostatic potential, e is the electron charge, $\hbar$ the reduced Planck constant and v is the velocity of the electrons used for imaging. Any magnetic contributions are disregarded. Eq. 1 is used to calculate the phase shift introduced by the mean-inner potential $V_{\mathrm{MIP}} = 28$ V of the thin gold islands,[2] as well as for the additional phase-shift due to the electrostatic potential induced by optical excitation. For the mean inner potential, Eq. 1 simplifies to $\Phi = e/(\hbar v^{*})V_{\mathrm{MIP}}t_{\mathrm{Au}}$ with t_{Au} as the thickness of the gold film.
For the contribution of the charging-related phase shift, the three-dimensional electrostatic potential is integrated along the z-axis. Details on the numerical calculation of the potential distribution are found in the following section.

From the wavefunction $\Psi(\vec{r})$ the resulting electron intensity on the detector $I(\vec{r})$ produced by an electron-optical system in Lorentz-mode can be calculated by

$$I(\vec{r}) = \left|\mathcal{F}^{-1}\left[T(\vec{q}_{\perp})\mathcal{F}\left(\Phi(\vec{r})\right)\right]\right|^{2}. \tag{2}$$

Here, $T(\vec{q}_{\perp})$ is the contrast transfer function defined in reciprocal space, with $\vec{q}_{\perp}$ as a wavevector in the plane perpendicular to the electron trajectory, and $\mathcal{F}$ as the Fourier transform in

the transverse plane. The contrast transfer function can be written as

$$T(\vec{q}_\perp) = e^{-i\chi(\vec{q}_\perp)} e^{-g(\vec{q}_\perp)} \tag{3}$$

in which $e^{-i\chi(\vec{q}_\perp)}$ is the phase-transfer function and $e^{-g(\vec{q}_\perp)}$ is a damping envelope incorporating a finite spatial coherence (temporal coherence effects are neglected). The parameters $\chi(\vec{q}_\perp)$ and $g(\vec{q}_\perp)$ are given by

$$\chi(\vec{q}_\perp) = \pi\lambda\Delta f|\vec{q}_\perp|^2 \tag{4}$$

$$g(\vec{q}_\perp) = \frac{(\pi\theta_c\Delta f)^2}{\ln 2}|\vec{q}_\perp|^2 \tag{5}$$

with Δf as the defocus of the imaging system and θ_c as beam divergence. The influence of spherical lens aberrations can be neglected due to the large defocus.[3] Using a defocus of $\Delta f = -10.5$ mm, the observed Fresnel fringes around the gold islands (without illumination) are well reproduced (see Fig. 1(b,d) in the main text). High-angle electron scattering within the gold islands and the small acceptance angle of the imaging system in Lorentz mode result in a decreased image intensity at the island position. We model this effect by considering an additional effective amplitude modulation $\sqrt{I_{mod}}$ for the electron wave components passing a gold island. The value for I_{mod} is extracted from in-focus images.

Calculation of the Electrostatic Potential

For calculating the electrostatic potential distribution V_{es} around the charged nanoislands, we adopt a numerical solution scheme for the corresponding Laplace equation in cylinder

coordinates[4]

$$\triangle V_{\mathrm{es}}\left(\rho,\phi,z\right) = \frac{1}{\rho}\frac{\delta}{\delta\rho}\left(\rho\frac{\delta V_{\mathrm{es}}}{\delta\rho}\right) + \frac{1}{\rho^2}\frac{\delta^2 V_{\mathrm{es}}}{\delta\phi^2} + \frac{\delta^2 V_{\mathrm{es}}}{\delta z^2} \tag{6}$$

$$= 0. \tag{7}$$

On the surface of the island the potential is constant. In the region surrounding the island, the electric potential can be approximated by considering a discretized version of the Laplace operator in the (ρ, z)-plane, utilizing the cylinder symmetry of the geometry. Thereby, the electrostatic potential $V_{\mathrm{es}}\left(\rho, z\right)$ at any position outside of the gold nanoislands needs to satisfy the relation

$$\begin{aligned} V_{\mathrm{es}}\left(\rho,z\right) =& \frac{1}{4}\big[V_{\mathrm{es}}(\rho+h,z) + V_{\mathrm{es}}(\rho-h,z) + V_{\mathrm{es}}(\rho,z+h) + V_{\mathrm{es}}(\rho,z-h)\big] \\ &+ \frac{h}{\rho}\big[V_{\mathrm{es}}(\rho+\frac{1}{2}h,z) + V_{\mathrm{es}}(\rho-\frac{1}{2}h,z)\big]. \end{aligned} \tag{8}$$

In order to find a function $V_{\mathrm{es}}\left(\rho, z\right)$ that satisfies this condition, an initial guess for V_{es} is placed on a equidistant 1024x1024 grid (effective pixel spacing 6.6 nm). This initial guess is relaxed towards a more accurate solution by changing the entries based on Eq. 8. The relaxation process is repeated for several iterations until the desired level of accuracy is achieved, while ensuring that the constraints imposed by the boundary conditions are satisfied. In our case the boundary conditions are a constant potential on the metallic island itself and vanishing potential at the box boundaries.

The electrostatic potential around a charged gold nanoisland was additionally calculated using the commercial finite-element simulation software "COMSOL". The result was in very good agreement with the electrostatic potential distribution as calculated by the successive over-relaxation method.

Extracting the light-induced voltage

To determine the light-induced voltage on the metallic islands, we use electron imaging simulations to reproduce the experimental data. Using a non-linear least squares algorithm, the image intensity $I(\vec{r})$ calculated by Eq. 2 is fitted to dark-corrected normalized and drift-corrected experimental micrographs by minimizing the squared intensity differences summed over all pixels. Here, the light-induced voltage U_{PV} on the metallic island is the only free fitting parameter.

As all mathematical operations in Eq. 8 are linear, the potential distribution does not have to be calculated for each value of U_{PV} in the fitting process.

Electron Micrograph for Tightly Focused Optical Illumination

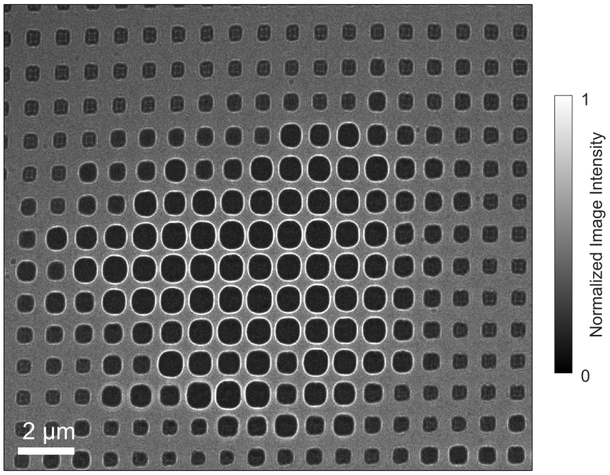

Figure S1: Defocused electron micrograph of an array of square-shaped gold nanoislands on a silicon nitride substrate. The center of the array is illuminated with high intensity light pulses (800-nm central wavelength, 190 fs pulse duration, 400 kHz repetition rate, p-polarized, average power: 1.2 mW). Different from the results in the main text, optical pulses were tightly focused to an area with 10 - 15 µm diameter.

Caption for Movie 1:

Assembled video showing defocused electron micrographs of optically excited gold nanoislands while scanning the pulse-to-pulse delay using a common-path birefringent interferometer.

Caption for Movie 2:

Assembled video of optically excited gold nanoislands recorded with an event-based electron detector based on a TimePix3 chip architecture. The video shows the reconstructed electron micrographs while chopping the optical excitation at a frequency of 5 Hz with a 50% duty cycle. We used an electron dose rate of 0.043 electron/(nm^2s).

Caption for Movie 3:

Assembled video of optically excited gold nanoislands recorded with an event-based electron detector based on a TimePix3 chip architecture. The video shows the reconstructed electron micrographs while chopping the optical excitation at a frequency of 5 Hz with a 50% duty cycle. We used an electron dose rate of 0.012 electron/(nm^2s).

Caption for Movie 4:

Assembled video of optically excited gold nanoislands recorded with an event-based electron detector based on a TimePix3 chip architecture. The video shows the reconstructed electron micrographs between consecutive light pulses with a repetition rate of 400 kHz. No change in image contrast is observable.

References

(1) Aharonov, Y.; Bohm, D. Significance of Electromagnetic Potentials in the Quantum Theory. *Phys. Rev.* **1959**, *115*, 485–491.

(2) Sanchez, A.; Ochando, M. A. Calculation of the Mean Inner Potential. *J. Phys. C: Solid State Phys.* **1985**, *18*, 33–41.

(3) De Graef, M. In *Magnetic Imaging and Its Applications to Materials*; De Graef, M., Zhu, Y., Eds.; Experimental Methods in the Physical Sciences; Academic Press, 2001; Vol. 36; pp 27–67.

(4) Hansen, P. B. Numerical Solution of Laplace's Equation. *Electrical Engineering and Computer Science - Technical Reports* **1992**, *168*.
